# Vitamin D enhances the efficacy of photodynamic therapy in a murine model of breast cancer

**DOI:** 10.1002/cam4.361

**Published:** 2015-02-25

**Authors:** Kishore R Rollakanti, Sanjay Anand, Edward V Maytin

**Affiliations:** 1 Department of Chemical and Biomedical Engineering, Cleveland State University2121 Euclid Avenue, Cleveland, Ohio, 44115; 2 Department of Biomedical Engineering, Cleveland ClinicCleveland, Ohio, 44195; 3 Department of Dermatology9500 Euclid Ave., Cleveland, Ohio, 44195

**Keywords:** Aminolevulinic acid, breast cancer, calcitriol, cutaneous metastasis, photodynamic therapy, vitamin D

## Abstract

Cutaneous metastasis occurs more frequently in breast cancer than in any other malignancy in women, causing significant morbidity. Photodynamic therapy (PDT), which combines a porphyrin-based photosensitizer and activation by light, can be employed for breast cancer (especially cutaneous metastases) but tumor control after PDT has not surpassed traditional treatments methods such as surgery, radiation, and chemotherapy up to now. Here, we report that breast cancer nodules in mice can be effectively treated by preconditioning the tumors with 1*α*, 25-dihydroxyvitamin D_3_ (calcitriol; Vit D) prior to administering 5-aminolevulinate (ALA)-based PDT. Breast carcinoma tumors (MDA-MB-231 cells implanted subcutaneously in nude mice) received systemic Vit D (1 *μ*g/kg) for 3 days prior to receiving ALA. The addition of Vit D increased intratumoral accumulation of protoporphyrin IX (PpIX) by 3.3 ± 0.5-fold, relative to mice receiving ALA alone. Bioluminescence imaging *in vivo* and immunohistochemical staining confirmed that tumor-specific cell death after ALA-PDT was markedly enhanced (36.8 ± 7.4-fold increase in TUNEL-positive nuclei; radiance decreased to 14% of control) in Vit D pretreated tumors as compared to vehicle-pretreated tumors. Vit D stimulated proliferation (10.7 ± 2.8-fold) and differentiation (9.62 ± 1.7-fold) in tumor cells, underlying an augmented cellular sensitivity to ALA-PDT. The observed enhancement of tumor responses to ALA-PDT after low, nontoxic doses of Vit D supports a new combination approach that deserves consideration in the clinical setting, and offers potential for improved remission of cutaneous breast cancer metastases.

## Introduction

Breast carcinoma is the most frequently diagnosed malignancy (except for skin cancer) and the second leading cause of cancer death in women in the United States; one in eight women (12%) are diagnosed with breast cancer during their lifetime, and >40,000 die annually from the disease 1. About 20% of women with breast carcinoma subsequently develop cutaneous metastasis (CM), a particularly painful form of locally recurrent cancer 2,3. In the setting of CM, the 5-year survival rate is 20%; median survival is 12–24 months 4. Patients with CM often develop resistance to systemic treatments including cytotoxic, hormonal, and immunotherapeutic agents 4. Ionizing radiation (IR) may work temporarily, but multiple IR treatments can induce secondary cancers 3. Due to the high morbidity and severe side effects (scarring, radiation dermatitis, etc.) with current methods, it is essential to develop new ways to treat breast cancer metastasis and achieve better efficacy with fewer complications.

One such emerging treatment modality is photodynamic therapy (PDT), which comprises three major components: a photosensitizing drug, a source of high-energy visible light, and tissue oxygen. To perform PDT, the drug is first administered, the photosensitizer is then allowed to accumulate selectively within tumor cells, and finally the tumor is illuminated with visible or near-infrared light to excite the photosensitizer; this ultimately results in formation of reactive oxygen species that destroy the tumor 5. PDT is widely used today to treat superficial lesions such as skin cancer and precancer, but PDT for internal cancers (with systemic delivery of drug, and light delivery through optical fibers) is still underutilized. Localized breast cancer was one of the first targets for PDT in the early studies by Dougherty using hematoporphyrin derivative 6, and PDT was subsequently shown to be effective in studies on patients with chest wall metastases 7–12. Using photofrin and red light, 18 of 37 patients (49%) achieved complete or partial responses in one study 9, complete responses in nine of 14 patients (64%) in another 12, and complete responses in 89% of 102 chest wall lesions in a third 11. Subsequent enthusiasm, however, has been tempered by the fact that prolonged skin photosensitivity is a major clinical side-effect with photofrin and other systemic photosensitizers.

In the early 1990s, PDT using 5-aminolevulinic acid (ALA) was introduced to provide more tumor specificity and less phototoxicity to normal tissues than with other types of PDT 13. ALA serves as a prodrug which is preferentially taken up by cancer cells and converted into an intracellular photosensitizer, protoporphyrin IX (PpIX), via eight enzymes in the heme biosynthetic pathway within mitochondria 14,15. ALA-PDT is effective for a number of malignancies, and has been investigated in breast cancer cell lines 16–18, mouse models of breast cancer 19, human breast tumor explants 20, and in clinical pilot studies 21–23. PpIX accumulates selectively in human breast cancer cells and tissues 19,21,22. Good PpIX levels can be achieved in patients with safe doses of oral ALA 21,22, and PpIX within tumor tissues can provide an effective therapeutic target in breast cancer 23. However, in breast cancer and in most other solid tumors, a persistent problem of insufficient PpIX levels and nonhomogeneous distribution of PpIX continues to result in incomplete or partial tumor responses to ALA-PDT 14.

Our laboratory has identified a number of cellular differentiation-promoting agents that increase the ability of epithelial cells to synthesize PpIX from exogenous ALA 14,24. Vitamin D is one such agent 25,26. In the current study, we investigated whether preconditioning with the active form of vitamin D (Vit D; calcitriol; 1*α*, 25-dihydroxyvitamin D_3_) can potentiate ALA-PDT in a breast cancer mouse model. Using bioluminescence imaging (BLI) to monitor tumor responses noninvasively 27–29 together with histological analyses, we show that Vit D pretreatment significantly enhances the efficacy of ALA-PDT for subcutaneously implanted breast cancer tumors. To our knowledge, we are the first to investigate the new treatment principle of differentiation-enhanced combination PDT in breast cancer, and we suggest that this combination may offer an effective approach for management of cutaneous breast cancer metastases that are accessible to surface illumination with red light.

## Materials and Methods

### Reagents

1*α*, 25-dihydroxyvitamin D_3_ (Vit D), and 5-aminolevulinic acid (ALA) hydrochloride were purchased from Sigma-Aldrich (St. Louis, MO). Luciferin (VivoGlo™ in-vivo Grade) was from Promega Corporation (Madison, MO).

### Cell culture

MDA-MB-231-luc-D3H2LN (MDA-Luc cells), a line of bioluminescent human adenocarcinoma breast cancer cells from Caliper Life Sciences (Hopkinton, Massachusetts, USA), was used within 6 months after resuscitation of the frozen vial. The commercial supplier authenticates cell lines and confirms they are pathogen-free, using the IMPACT Profile I (PCR) at the University of Missouri Research Animal Diagnositc and Investigative Laboratory. Cells were cultured at 37°C in MEM medium with Earle's salts and 10% fetal bovine serum (FBS; BioWhitaker, Walkersville, MD) and 1% penicillin-streptomycin. Subconfluent cells were passaged using 0.05% trypsin-53 mmol/L EDTA solution, followed by addition of FBS-containing medium. Cells were used at or before passage 6.

### Murine breast cancer model

Immunocompromised nude female mice (*nu/nu*, 4-week-old), purchased from Charles River Laboratories (Wilmington, MA), were injected subcutaneously with ∼5 × 10^5^ MDA-Luc cells in Matrigel (BD Biosciences, San Jose, CA) and MEM medium in a 1:1 ratio (4°C), into the breast fat pads. Animal care activities and protocols were approved and monitored by our Institutional Animal Care and Use Committee (IACUC).

### *In-vivo* BLI

**Figure 1:**
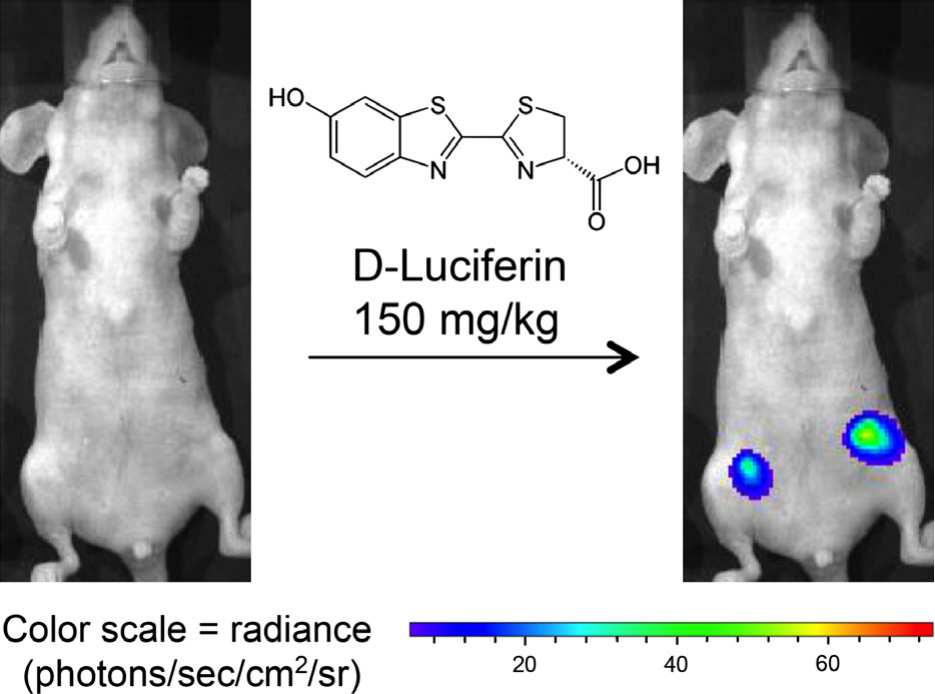
Noninvasive bioluminescence monitoring of MDA-MB-231-luc breast cancer cells in the subcutaneous fat pads of nude mice using the IVIS Imaging System.

IVIS® 100, a commercial BLI system (PerkinElmer, Inc. Waltham, MA) was used to acquire luminescent images from breast cancer-bearing mice *in-vivo*. Images were collected; (1) at 72 h after cancer cell implantation, (2) just prior to ALA injection, and (3) at 24 h after PDT. Prior to *in-vivo* imaging, mice were anesthetized with inhaled 1–3% isoflurane. They were then given d-luciferin (150 mg/kg subcutaneously, in Dulbecco's phosphate buffered saline, PBS) and placed on a warm (37°C) stage inside the light-tight camera box, under continuous 1–3% isoflurane anesthesia. Three or four mice were imaged at the same time. Camera settings were: Field of View, B; Exposure time, 20 sec; Binning, medium; F/stop, 1. Digital images were obtained 12–15 min after d-Luciferin injection. Images from multiple days were normalized and analyzed using LivingImage® 4.2 software (Xenogen, Alameda, CA, USA); the emitted light in each region of interest (ROI) was quantified as total photon counts, or radiance (see Fig.1).

### Preconditioning with Vit D

Beginning at 3 days post implantation, tumors were preconditioned with Vit D (calcitriol, 1 *μ*g/kg intraperitoneal (i.p.) injection in PBS) once daily for 3 days.

### Analysis of intracellular protoporphyrin IX (PpIX)

On day 4, after 3 days of Vit D preconditioning, ALA (200 mg/kg in PBS) was delivered by i.p. injection. Mice were sacrificed 4 h later, tumors harvested, and the tissue embedded in O.C.T. medium (Tissue-Tek; Sakura-Finetek, Torrance, CA, USA) for frozen sectioning. PpIX-specific fluorescence (*λ*_ex_ 633 nm; *λ*_em_ 650–780 nm) from 10 *μ*m thick cryosections was observed by confocal microscopy (Leica Microsystems, Buffalo Grove, IL) and quantitfied using IPLab image processing software (Signal Analytics, Vienna, VA) as previously described 30.

### PDT treatment

At 4 h after ALA injection, mice were irradiated at a fluence rate of 0.43 W/cm^2^, using a 633 nm noncoherent light source (LumaCare USA, Newport Beach, CA) calibrated with a FieldMate laser power meter (Coherent Inc., Santa Clara, CA). Each tumor received 250 J/cm^2^ of 633 nm light over ∼10 min.

### Histology and immunohistochemistry

After completion of bioluminescence measurments, mice were euthanized, tumors excised, fixed in formalin, and paraffin-embedded. Histological sections (5 *μ*m) were analyzed by hematoxylin and eosin (H&E) staining, terminal dUTP nick end labeling (TUNEL) (Roche Applied Science, Indianapolis, IN), E-cadherin (Santa Cruz Biotechnology, Santa Cruz, CA), and Ki-67 immunostaining (Neomarkers Inc., Fremont, CA) per the manufacturer's directions. Digital images were obtained using a Leica SCN400 system, and the H&E or immunofluorescently stained specimens were analyzed using IP Lab image processing software, as described earlier 30.

### Statistical analyses

Two-sample *t*-tests were used to compare differences in PpIX accumulation, mean bioluminescence, and other IHC staining data between different treatment groups. *P* ≤ 0.05 were considered statistically significant.

## Results

### Vit D preconditioning increases PpIX synthesis in MDA-MB-231-luc tumors

**Figure 2:**
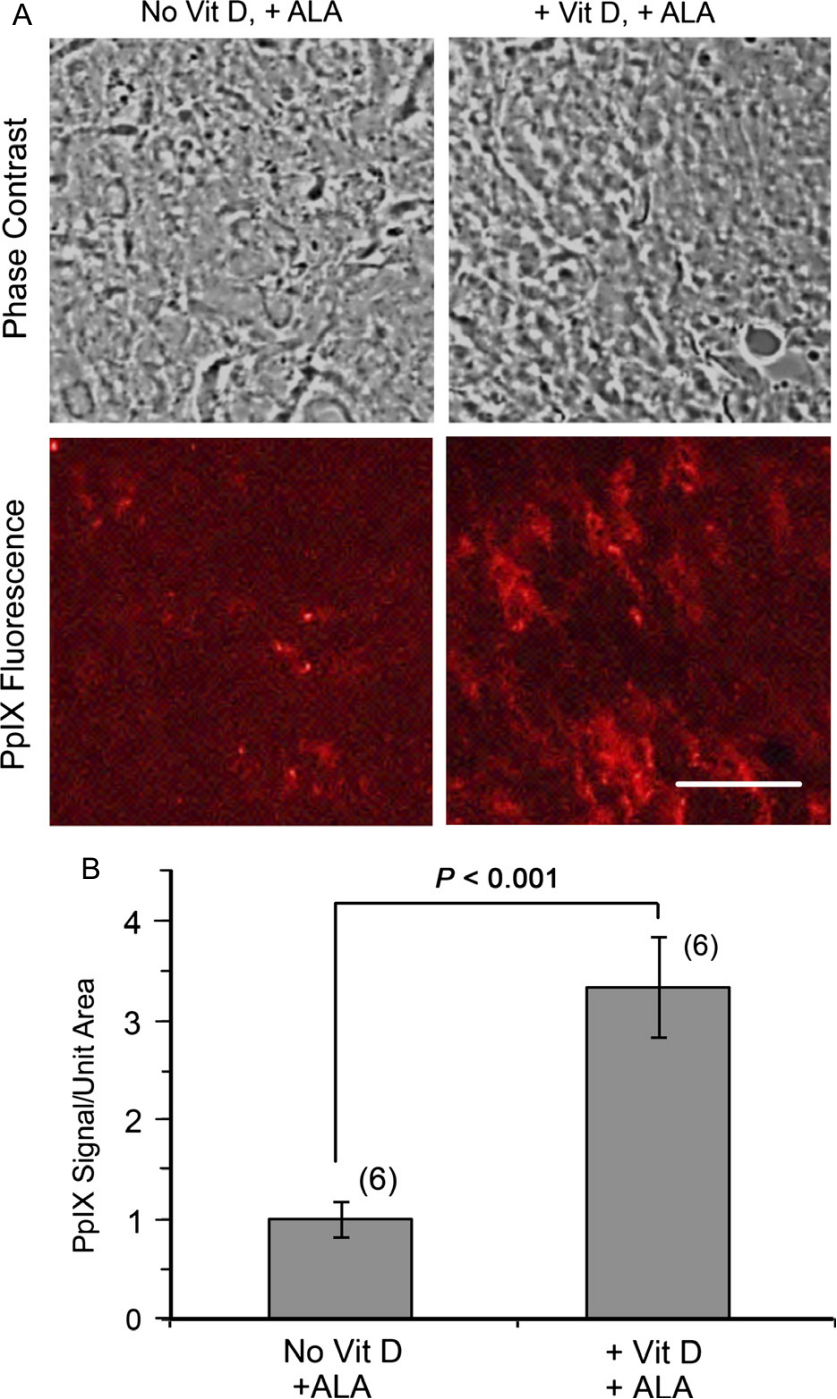
Effect of Vit D preconditioning on PpIX synthesis. (A) Confocal images of PpIX in frozen tumor sections after 3 days of saline or Vit D pretreatment followed by ALA on day 4. Scale bar, 25 *μ*m. (B) Quantification of PpIX-specific fluorescence in the digital confocal images. Mean ± SEM, six tumors per condition; *P* values from unpaired two-sided *t*-tests. ALA, 5-aminolevulinate.

As illustrated in Figure2A, confocal images showed greater intensity of PpIX-specific fluorescence in frozen sections from Vit D preconditioned tumors than in tumors from the saline-treated group. Digital quantification of the fluorescent signal confirmed this result (Fig.2B), showing a statistically significant increase (3.3 ± 0.5-fold) in the ALA-induced intracellular PpIX level with Vit D treatment. Compared to doses of Vit D typically used in oncology, the dose of Vit D used here (1 *μ*g/kg) is very low and was shown to be safe and nontoxic in recent studies in other mouse tumor models 25,31.

### Noninvasive monitoring of tumors suggests that Vit D pretreatment enhances tumor cell death after ALA-PDT

**Figure 3:**
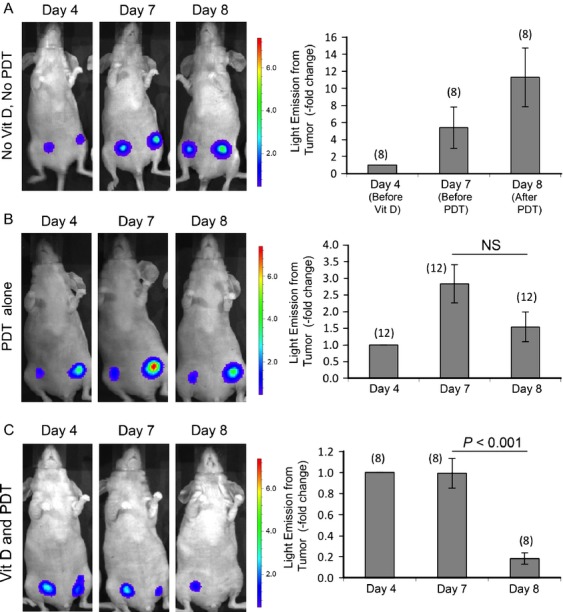
Noninvasive monitoring of treatment responses in breast cancer tumors. Photographs illustrate typical changes in individual mice; BLI signal superimposed upon white light image. (A) Control mouse that received neither Vit D treatment nor ALA-PDT; (B) mouse that received only ALA-PDT; (C) mouse that received both Vit D treatment for 3 days followed by ALA-PDT on fourth day. Graphs quantify relative change in radiance (photons sec^−1^ cm^−2^ sr^−1^). Mean ± SEM; number of tumors per treatment group in parenthesis. *P* values from paired two-sided *t*-tests. *NS*, not significant; BLI, bioluminescence imaging; ALA, 5-aminolevulinate; PDT, photodynamic therapy.

Figure3 shows representative examples of the BLI signal from orthotopic breast tumors *in-vivo* at three different times in various experimental groups, before and after PDT. The bioluminescence signal, displayed as a pseudocolored scale, corresponds to the radiance (photons sec^−1^ cm^−2^ sr^−1^) emitted from each tumor due to the reaction between luciferin substrate and luciferase enzyme. In the first group of mice which received no agents (Fig.3A), radiance continued to increase from day 4 to day 8, consistent with continuous/unrestricted growth of luciferase-bearing cancer cells in the breast fat pad environment. In the second group of mice, the increase in radiance between day 7 and day 8 was reversed by application of ALA-PDT at day 7, resulting in a 45% drop in signal (Fig.3B). However, radiance from these tumors at day 8 was still higher than at day 4, indicating room for improvement in the treatment protocol. In contrast, all animals that received Vit D/ALA-PDT treatment showed a statistically significant reduction (82 ± 6%, *P *<* *0.001) in radiance at day 8, suggesting that viable tumor cells had decreased to one-fifth of the number before PDT (Fig.3C). (To confirm that the decrease in luminescence after VitD/ALA-PDT actually corresponded to changes in the number of living cells after treatment, tumors were histologically examined after biopsy; see below).

### Vit D stimulates differentiation and proliferation in MDA-MB-231-luc tumors

**Figure 4:**
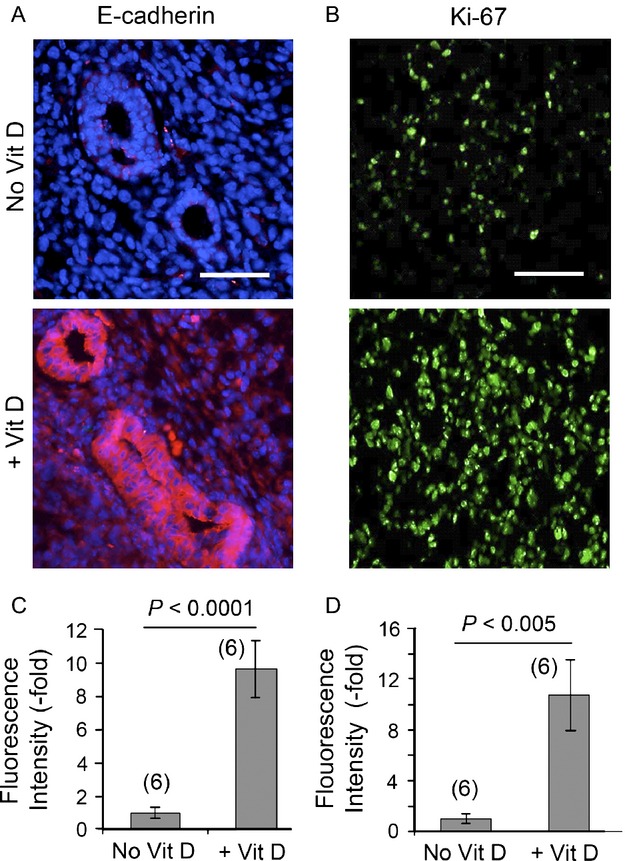
Vit D preconditioning increases the status of proliferation and differentiation in MDA-MB-231-luc tumor cells. Images show paraffin sections of the tumors, immunostained with antisera to (A), E-Cadherin (red fluorescence), and (B), Ki-67 (green fluorescence). Quantification of relative staining intensity of (C), E-Cadherin and (D), Ki-67 from multiple histologic specimens of the tumors. Scale bars, 50 *μ*m. Mean ± SEM from six tumors per condition. The *P* values from unpaired two-sided *t*-tests are indicated.

To examine the effect of Vit D pretreatment on differentiation and proliferation status, tumor tissues were stained for E-cadherin and Ki-67, respectively. When compared with saline-treated control tumors, E-cadherin expression was significantly increased in Vit D pretreated tumors (Fig.4A). E-cadherin was mainly expressed in cells within ductal structures in the tumors that received Vit D, whereas E-cadherin in saline-treated tumors was undetectable (Fig.4A). Expression of nuclear Ki-67 was also induced, being observed in the majority of cells within tumors receiving Vit D preconditioning (Fig.4B). Quantitatively, E-cadherin and Ki-67 were increased 9.6 ± 1.7-fold and 10.7 ± 2.8-fold, respectively, in Vit D-preconditioned tumors relative to nonconditioned controls (Fig.4C and D).

### Vit D preconditioning increases ALA-induced phototoxicity

**Figure 5:**
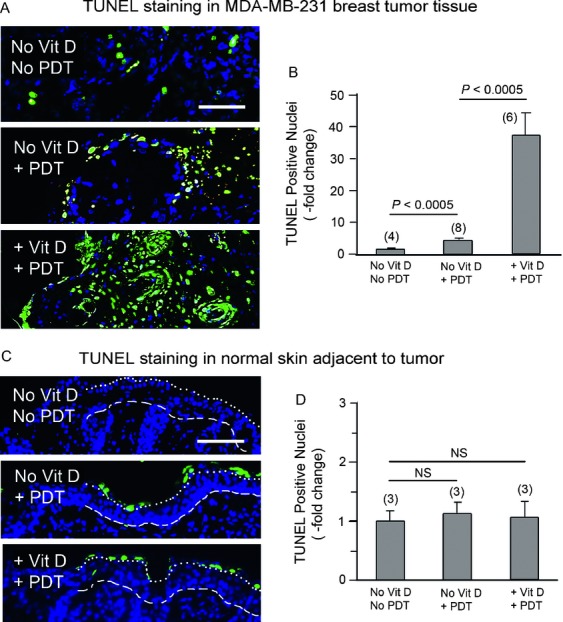
Vit D pretreated breast cancer shows enhanced tumor-specific cell death after ALA-PDT. (A) Examples of TUNEL-labeled apoptotic nuclei in tumors from control (top), vehicle-PDT (middle), and Vit D-PDT (lower) groups. (B) Quantification of cell death in tumors, treated as in (A). (C) Cell death in normal skin adjacent to the tumors in control (top), vehicle-PDT (middle), and Vit D-PDT (lower) groups. (D) Quantification of cell death in the normal epidermis shows no significant differences. Dotted line, junction between stratum corneum (above) and viable epidermis. Dashed line, basement membrane between dermis and epidermis. Data points represent mean ± SEM; *P* values from unpaired two-sided *t*-tests; numerals in parenthesis indicate number of tumors (three histologic sections per tumor) for each treatment condition. Scale bars, 50 *μ*m. ALA, 5-aminolevulinate; PDT, photodynamic therapy.

To assess cell-death responses after treatment, a TUNEL assay was performed on formalin-fixed/paraffin-embedded tissues (Fig.5). Results indicated a greater amount of cell death in the MDA-MB-231-luc tumors preconditioned with Vit D prior to ALA-PDT, relative to ALA-PDT alone. Very few TUNEL-positive cells were observed in absolute control tumors (Fig.5A, top). After ALA-PDT, only scattered cell death was seen, limited to the peripheral region of the tumors (Fig.5A*,* middle). In contrast, widespread cell death was observed throughout tumors that were pretreated with Vit D prior to ALA-PDT (Fig.5A, lower). Quantitative TUNEL results indicated a 4.0 ± 0.7-fold increase in apoptotic cells after ALA-PDT alone, versus a 36.8 ± 7.4-fold increase in tumor cell death after combined treatment (Fig.5B), a highly significant difference.

Importantly, skin at locations adjacent to the tumor (and exposed to the same treatment) showed a very low number of TUNEL-positive cells, and was statistically indistinguishable between the experimental conditions (Fig.5C and D). Although a few TUNEL-positive cells were seen in the stratum corneum (above the epidermis), none were seen in the viable epidermal layer. These results confirm a high degree of tumor-selectivity after ALA-PDT using the combined treatment.

### H&E staining confirms the increased efficacy of the combination approach

**Figure 6:**
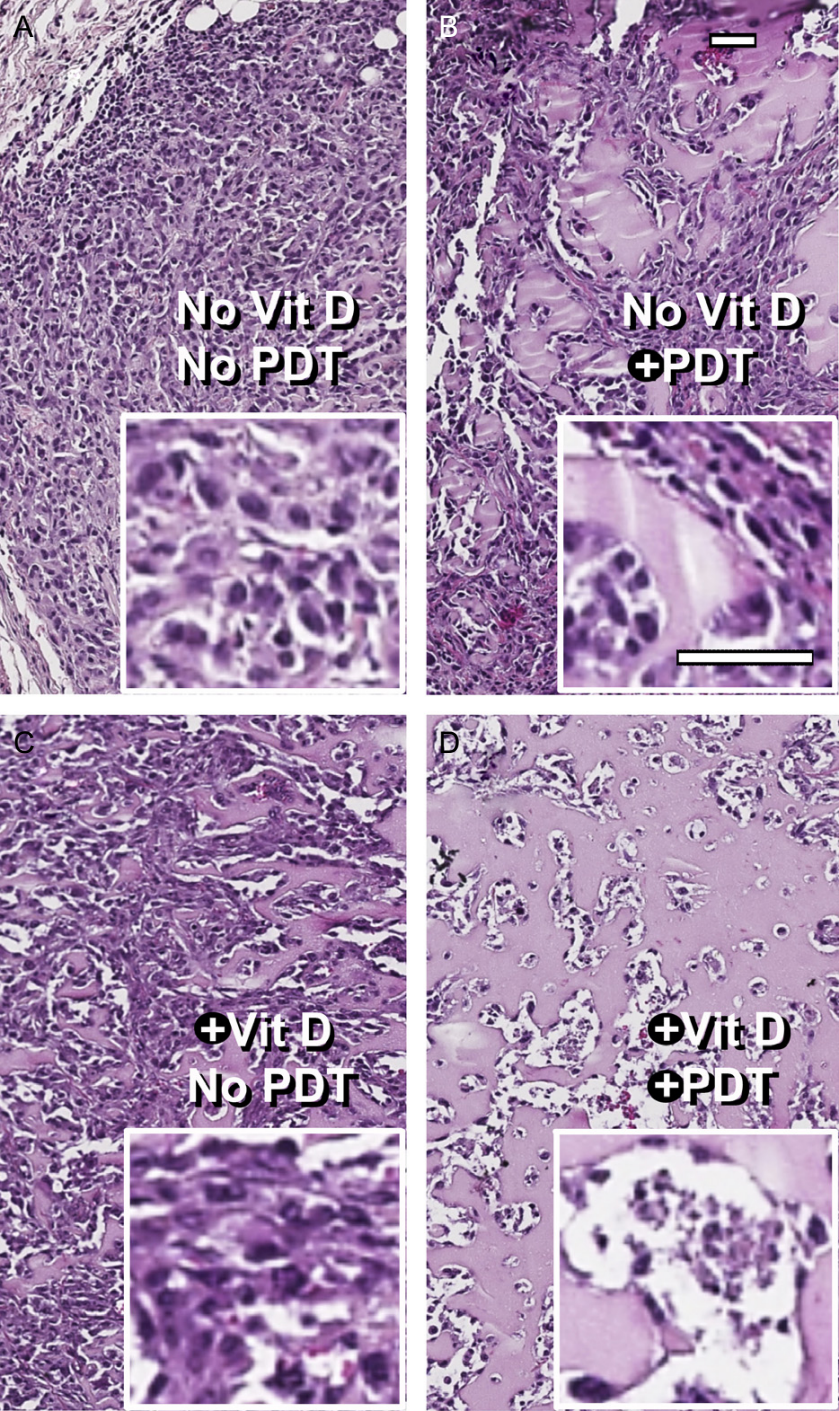
Morphological differences in tumors subjected to various treatment conditions. (A–D), These images illustrate the progressive loss of nucleated cells, the increase in colloid-filled cystic spaces (pink), and the increase in completely empty spaces (white), occuring with the addition of Vit D to the ALA-PDT treatment regimen. Scale bars, 50 *μ*m. ALA, 5-aminolevulinate; PDT, photodynamic therapy.

**Figure 7:**
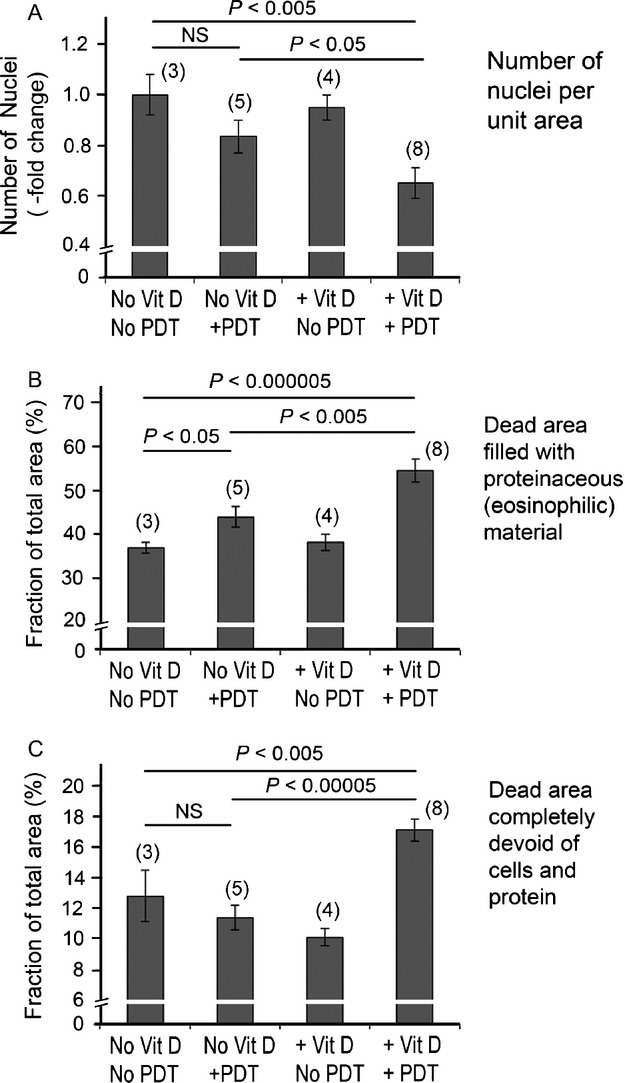
Quantification of different parameters in H&E-stained tumor images. Graphs (A), (B), and (C) represent the number of nuclei, pink space, and white space per region of interest (ROI), respectively. All data points represent mean ± SEM; *P* values from unpaired two-sided *t*-tests. Parentheses, number of tumors evaluated (three sections per tumor). *NS*, not significant.

**Figure 8:**
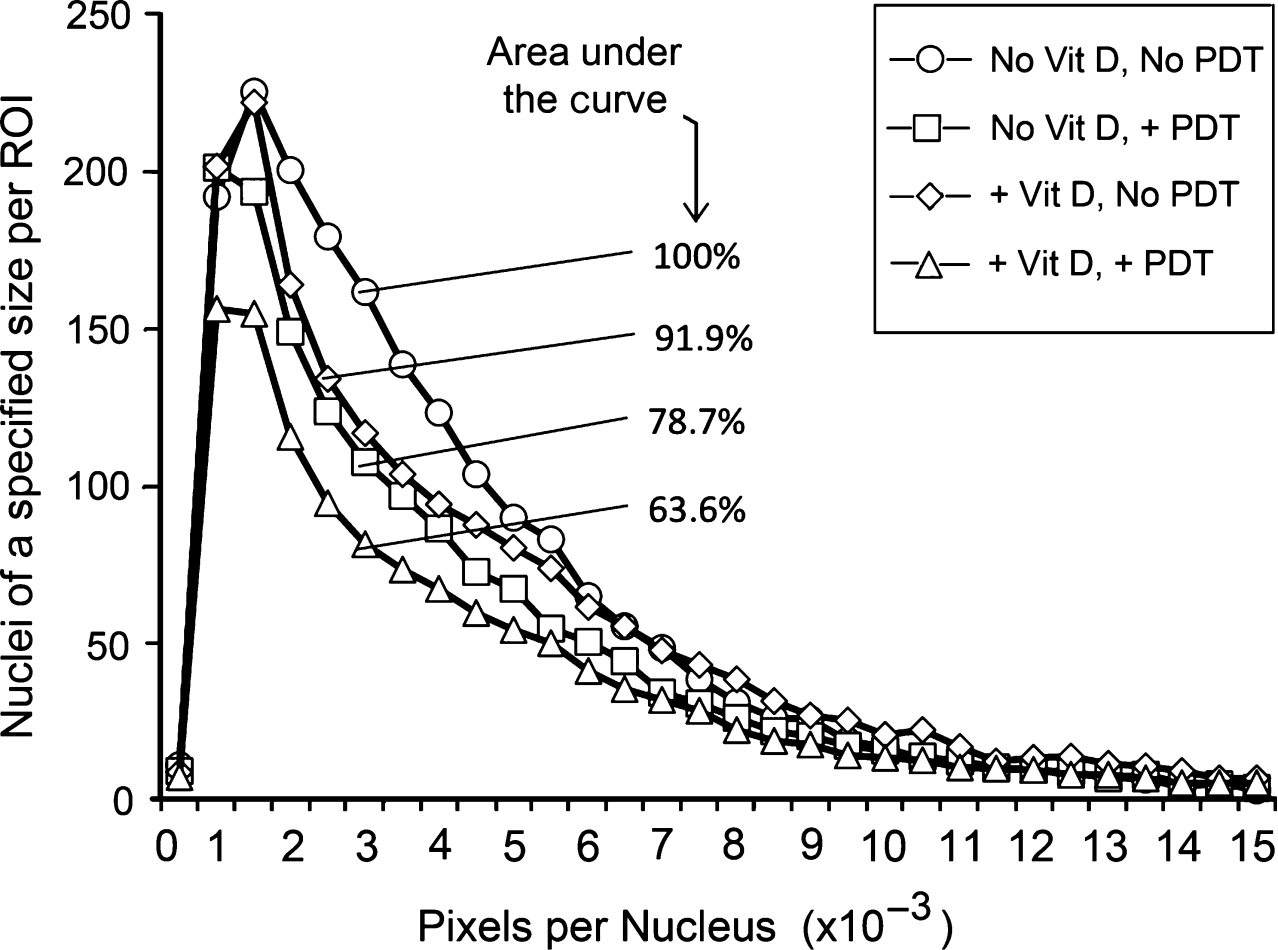
Histogram of nuclear sizes in tumors after different treatments. Nuclear sizes were determined from image processing and divided into bins; markings on the histogram indicate number of nuclei in each corresponding bin (or size range). Percentages indicate area under the curve (AUC) relative to the vehicle.

Figure6 shows representative images of H&E stained sections of paraffin-fixed tumors from four different treatment groups. Untreated controls (Fig.6A) were characterized by the presence of healthy cells arranged in lobular or ductal patterns, which filled the entire microscopic field. In tumors treated with ALA-PDT alone (Fig.6B), and to some extent with Vit D alone (Fig.6C), loss of nucleated cells was observed. However, tumors that received Vit D/ALA-PDT (Fig.6D) showed a dramatic decrease in the density of nucleated cells as compared to control tumors (Fig.7A). In addition, a greater fraction (60–70%) of the tumor area was occupied by eosinophilic material, most likely colloidal casein or other proteins (Fig.7B) or empty spaces completely devoid of any material (Fig.7C). As an additional measure of dying cells, pixel-by-pixel size analysis of nuclei was performed, revealing the highest number of shrunken (pyknotic) or fragmented nuclei in the Vit D-ALA-PDT group (Fig.8). To create a semiquantitative index (damage severity index, DSI) that incorporates all these features and correlates with PDT-induced damage, parameters from Fig.7 were combined with the nuclear size analysis (Fig.8), and the sum of all parameters used as a histologic index (Table1). Values of this index were four-fold higher after Vit D plus ALA-PDT than after ALA-PDT alone, further corroborating the results obtained from bioluminescence (Fig.3) and TUNEL (Fig.5A).

**Table 1:** Damage severity index (DSI)

Treatment group	% decrease in number of nuclei/ROI^1^	% increase in pink space^2^	% increase in white space^3^	% decrease in area occupied by nuclei^4^	DSI = sum of columns 1 to 4
Vehicle	0	0	0	0	0
Vit D alone	5	3.5	−21	21.3	8.8
Vehicle + PDT	17	19.1	−10.9	8.1	33.3
Vit D + PDT	35	47.7	33.6	36.4	152.8^5^

Four different histological parameters that quantify different aspects of tissue damage in breast tumors at 24 h after ALA-PDT, are shown in the table. The sum of these four parameters is the DSI. PDT, photodynamic therapy.

1 Number of nucleated cells as counted by IP lab software (see Fig.6).

2 Space filled with eosinophilic material such as casein or other proteins.

3 Space completely devoid of any material.

4 Values obtained from the size distribution of nuclei shown in Figure8.

5 Fourfold more than vehicle + PDT.

## Discussion

We have developed a new combination approach that uses Vit D to sensitize breast cancer cells to ALA-PDT. Using bioluminescence with histological confirmation, we have shown that tumor nodules in mice can be treated more effectively with a Vit D/ALA-PDT combination than with ALA-PDT alone. Vit D preconditioning enhances the proliferation and differentiation status of the tumor cells, and increases ALA-driven PpIX production, with a cumulative result of significantly higher tumor cell death with the combination than with ALA-PDT alone.

Different approaches, used together, strengthen the reliability of our findings. Bioluminescence is a functional assay that requires living, *luc*-transfected cells to support a photochemical reaction between luciferin and luciferase 32,33, and therefore the loss of signal after VitD/ALA-PDT treatment appeared to reflect a loss in viable cell number. This assumption, bolstered by reports of a strong correlation between mean tumor volume and bioluminescence in previous *in-vivo* studies with the MDA-MB-231-luc cell line 34, was confirmed by histological analyses of biopsied tissue. Dying tumor cells exhibited DNA fragmentation (Fig.5), H&E staining appearance (Fig.8), and nuclear shrinkage and fragmentation, all consistent with enhanced cell destruction after combined Vit D/ALA-PDT treatment (Fig.8).

Our data, showing that Vit D induces synthesis of PpIX (the major intracellular photosensitizer during ALA-PDT), and stimulates proliferation and differentiation of breast tumor cells, suggest potential mechanisms for how Vit D might potentiate ALA-PDT-mediated cell death. PpIX is produced by enzymes of the heme synthetic pathway 35; Vit D changed the levels of two enzymes, coproporphyrin oxidase and ferrochelatase, in a way that favors accumulation of PpIX in another epithelial tumor model (subcutaneous A431 cells in mice) 25, and a similar mechanism might be operative here. Importantly, Vit D enhances ALA-PDT in a tumor-specific manner, causing minimal damage to normal skin (Fig.5), although the exact mechanisms for this selectivity remain to be investigated. Differentiation of breast tumor cells is stimulated by Vit D, but how this might contribute to PDT sensitivity is an open question. By analogy, breast cancer cells become more chemosensitive when forced to differentiate into specific lineages by treatment with cisplatin 36,37, but the mechanism here may be different. Differentiation status and tumor sensitization to ALA-PDT are strongly correlated, now observed in ALA-PDT combination regimens involving methotrexate 30, 5-fluorouracil, and Vit D 25, and involving transcriptional changes in gene expression 38. Regarding proliferation, Vit D at pharmacological doses usually causes growth arrest, but the situation here is different, in which extremely low doses of Vit D (picomolar range in tissue) stimulate cell proliferation and effectively enhances ALA-PDT, as shown previously 25,26. As hypothesized by Wyld, in cells pushed to actively divide (by Vit D in this case) the DNA may become more susceptible to damage by the photodynamic treatment 39.

Our preclinical findings have implications for the future development of ALA-PDT for cutaneous breast cancer in terms of (1) improved efficacy, (2) safety, and (3) acceptability to patients. Improved efficacy will be critical. As shown here and in previous studies 14,25, PpIX accumulation is usually spotty (nonhomogenous) in tumors so that many PpIX-negative cells manage to escape the photocytotoxic effects of PDT. By elevating the PpIX level in the majority of tumor cells, Vit D makes the entire tumor an effective target for ALA-PpIX mediated killing. Vit D pretreatment may also increase the effective depth for light penetration (currently limited to <5 mm deep with red light sources), since more cells can accumulate PpIX concentrations that exceed the threshold for phototoxic killing. Drug delivery per se should not be an issue; ALA has a well-established pharmacokinetic profile after oral delivery 40–42 and provides easily detectable PpIX levels within breast tumors in patients 3–6 h after ALA ingestion 21,22. Oral delivery of ALA also appears to be very safe; the only concern is skin photosensitivy for 24–36 h after ingestion, requiring strict sun avoidance. Vit D on the other hand is a hormone that can cause hypercalcemia. At the very low doses of Vit D used here, the potential for hypercalcemia is exceedingly low, based upon data from two other recent recent studies using the same dose of Vit D (1 *μ*g/kg) in mice 25,31. Also, Vit D is used as a short pulse (only three daily doses) to prime the tumor cells for ALA-PDT, reducing the risk further. Aiming to eliminate the risk of hypercalcemia entirely, recent experiments suggest that cholecalciferol (the natural dietary form of Vit D) may work as a substitute to calcitriol in combination with ALA-PDT in a mouse model of squamous cell carcinoma (SCC) 31, which would be intriguing approach to test for breast cancer. Regarding patient acceptance of Vit D/ALA-PDT for chest wall metastases, any alternative that works would be greatly appreciated in patients whose disease has become resistant to salvage surgery, chemohormonal therapy, and ionizing radiation. ALA-PDT, unlike surgery, is minimally invasive and causes little to no scarring. Unlike ionizing radiation, ALA-PDT can be performed repeatedly without increasing the risk for oncogenic mutations (since PDT damages mitochondrial membranes, not DNA). A recent trial showed that ALA-PDT, combined with radiation therapy, can improve the efficacy of the latter is already a step in the right direction 23. Vit D/ALA-PDT might eventually prove to be an excellent alternative for patients in whom other treatments have failed, either alone or in combination with other salvage paradigms.
